# A systematic study of genome context methods: calibration, normalization and combination

**DOI:** 10.1186/1471-2105-11-493

**Published:** 2010-10-01

**Authors:** Luciana Ferrer, Joseph M Dale, Peter D Karp

**Affiliations:** 1 Artificial Intelligence Center, SRI International, Menlo Park, California, USA

## Abstract

**Background:**

Genome context methods have been introduced in the last decade as automatic methods to predict functional relatedness between genes in a target genome using the patterns of existence and relative locations of the homologs of those genes in a set of reference genomes. Much work has been done in the application of these methods to different bioinformatics tasks, but few papers present a systematic study of the methods and their combination necessary for their optimal use.

**Results:**

We present a thorough study of the four main families of genome context methods found in the literature: phylogenetic profile, gene fusion, gene cluster, and gene neighbor. We find that for most organisms the gene neighbor method outperforms the phylogenetic profile method by as much as 40% in sensitivity, being competitive with the gene cluster method at low sensitivities. Gene fusion is generally the worst performing of the four methods. A thorough exploration of the parameter space for each method is performed and results across different target organisms are presented.

We propose the use of normalization procedures as those used on microarray data for the genome context scores. We show that substantial gains can be achieved from the use of a simple normalization technique. In particular, the sensitivity of the phylogenetic profile method is improved by around 25% after normalization, resulting, to our knowledge, on the best-performing phylogenetic profile system in the literature.

Finally, we show results from combining the various genome context methods into a single score. When using a cross-validation procedure to train the combiners, with both original and normalized scores as input, a decision tree combiner results in gains of up to 20% with respect to the gene neighbor method. Overall, this represents a gain of around 15% over what can be considered the state of the art in this area: the four original genome context methods combined using a procedure like that used in the STRING database. Unfortunately, we find that these gains disappear when the combiner is trained only with organisms that are phylogenetically distant from the target organism.

**Conclusions:**

Our experiments indicate that gene neighbor is the best individual genome context method and that gains from the combination of individual methods are very sensitive to the training data used to obtain the combiner's parameters. If adequate training data is not available, using the gene neighbor score by itself instead of a combined score might be the best choice.

## 1 Background

In recent years, large-scale genome sequencing has resulted in a steep growth in the number of fully sequenced genomes. Part of the sequencing effort is to automatically annotate the genome with structural information (for example, location of open reading frames and coding regions) and functional information. Much of this annotation process relies on finding homologs of the target genes in other annotated genomes. The target gene often inherits the function of its homologous sequences, when available. Using this method, genes that do not have an annotated homologous sequence in any other genome cannot be assigned a function.

### Genome Context Methods

Genome context analysis denotes a family of techniques used to infer functional relationships between genes using a comparative analysis approach that allows for the inference of function across genes that may not share sequence similarity. These techniques are based on assumptions drawn from knowledge about evolutionary processes. For example, the phylogenetic profile method [1] uses the patterns of occurrence of a gene across a set of genomes. Two genes with similar occurrence patterns are likely to be functionally related. The assumption is that organisms are under evolutionary pressure to encode either both genes or neither gene if the genes are related. Other genome context techniques use evidence such as protein fusions [2-4], proximity of genes within the genome [4,5], and proximity of homologous sequences of the genes across a list of reference genomes [4]. These methods will be explained in detail in Section 2.

Many other sources of information have been used for functional prediction (see, for example, [6,7]), including mRNA co-expression data, MIPS functional similarity, GO functional similarity, co-essentiality, and co-regulation. All these extra sources of information rely on data other than that available in the annotated genome sequence. In this paper, we limit our study to features that can be extracted automatically from the annotated genome.

### Normalization of Genome Context Scores

Genome context methods generate numeric values, or *scores*, for pairs of genes. These scores are assumed to be correlated with the probability that the two genes are functionally related. Unfortunately, scores, in most cases, indirectly capture other characteristics of the two genes involved. For example, measures of similarity between two phylogenetic profiles might be affected by how frequent the genes are in the list of genomes used to compute the profiles. This bias in the scores can degrade the performance of genome context methods. The problem of score bias is, in fact, a common problem across many statistical processing problems. A well-known example of data that suffers this problem in bioinformatics is microarray data. In this case, measurements coming from different microarrays are affected not just by the differences we intend to study, but also by differences in the scanner or in the production of the array. Normalization procedures are designed to compensate for this problem [8,9]. In this paper, we adapt two normalization procedures that have been used for the microarray problem to the problem of estimating functional relations from genome context scores. We demonstrate varying degrees of relative gain in sensitivity of as much as 40% depending on the type of score, at operating points corresponding to high specificity.

### Combination of Genome Context Scores

The different genome context scores implemented in this paper capture somewhat different information about the samples. Hence, one would expect that combining these scores into a single score should lead to a score that is better than any of the individual ones. Combination of genome context scores has been explored in many papers [4,7,10-15]. Two related methods are used in the STRING database [14,16] and the Prolinks database [4]. In both cases, scores are first individually transformed into *confidence measures *using a labeled training set. The resulting confidence measures are then combined into a single measure by picking the maximum [4] or using a simple product expression [14]. The same product expression is used in [12], but in this case the individual scores are first weighted by a factor that depends on the performance of the method. In all these cases, the authors present the results of the combination on the same data used to train the transform into confidence measures or find the weights for the different methods. That is, the parameters of the combination are the optimal parameters on the data where results are being reported. This results in an optimistic prediction of the gains that can be achieved from combination. It is not clear from these experiments whether such results would generalize to unseen data.

More complex combination procedures have also been proposed. In [11] a support vector machine is trained to combine three different genome context method scores, showing that the combination outperforms the individual methods (both sensitivity and specificity are improved) when the combiner is trained with cross-validation on *Escherichia coli *gene pairs. That is, gene pairs are randomly split into sets and each set is classified using the combiner trained on all remaining sets. Combination results including many other information sources apart from genome context methods (for example, mRNA co-expression data and GO functional similarity) are presented in [7]. In this case, the combiner is also trained using cross-validation, although the dataset includes gene pairs from the complete MIPS catalog, not from a single organism. Results show a modest gain in performance from combination, although none of the scores that are used in the final combination correspond to genome context scores.

Some of the papers mentioned above (for example, [10-12]) present the performance of the systems after a hard decision on the label of each sample has been made. Sometimes this is done because the system directly outputs a binary decision. At other times, the hard labels are obtained by thresholding of a continuous score with some predetermined threshold. In either case, comparing systems that make hard decisions is not straightforward, since, generally, systems end up at different operating points. If neither the false positive nor false negative rate of two systems is the same, there is no direct way to compare them, unless one of the systems has a strictly smaller value for both measures, in which case it can be declared better than the other (this is the case for some results in the papers cited above). When that is not the case, a performance measure that combines the two types of errors is generally used to compare the systems. Choosing such a performance measure implies a rather arbitrary decision on the costs of the different types of errors.

Hence, in our judgment, not enough strong evidence on the *degree *of improvement that can be obtained from combination procedures for genome context scores has yet been presented in the literature. Furthermore, to our knowledge, no attempt has yet been made at training combination procedures for genome context methods on certain organisms to apply them on other organisms not included in the training set. This is, arguably, the most realistic scenario where the goal is to assign functional relatedness labels to a new organism for which no or little manual curation has yet been done and for which no related strain has been curated either. In such a case, cross-validation or train-on-test results would not be applicable. In this paper, we present comparative results of combination performance when training the combiner on organisms that are phylogenetically distant to the test organism and using cross-validation on the test organism.

### Parameter Tuning for Genome Context Methods

In addition to introducing the normalization procedure for genome context scores, and showing several combination results, we present a large set of experimental results exploring the parameter space of the different methods. Some experimental studies on the optimal settings of the parameters of the genome context methods have been presented in the literature. Sun et al. [17] explore the performance of the phylogenetic profile method when the distance metric is given by mutual information for different values of the BLAST E-value threshold and different composition of the reference list. These same parameters are explored in [18], along with the metric used to compute the distance between phylogenetic profiles. Cokus et al. [19] also explore different metrics, some of them rather sophisticated, and propose a new one that outperforms all other metrics tried in their paper. To our knowledge, optimization studies of this type have not been performed for genome context methods other than phylogenetic profile.

In this paper, we thoroughly explore the parameter space of each method, including the percent similarity required in the gene fusion method and the E-value threshold used to infer homology. Furthermore, six different metrics of phylogenetic profile similarity are explored, including the metric proposed by Cokus et al. [19]. We also include a study of the effect of the size and composition of the list of reference genomes on all methods and a comparison of results of the different methods across different organisms. We show most results in terms of receiver operating characteristic (ROC) curves, which allow us to compare systems without committing to a certain set of costs for the different types of errors.

### Summary of Contributions

The contributions of this paper are (1) a normalization procedure for the genome context scores that improves performance of the mutual information phylogenetic profile method by around 25%, resulting, to our knowledge, on the best-performing phylogenetic profile method to date; (2) a thorough exploration of the effect on performance of the parameters of the different genome context methods; (3) a comparison of performance of the different methods on a set of bacterial organisms, from where we observe a variation of a factor of 2 in the sensitivity achieved by the different methods across organisms, and a rather consistent ranking of the different methods with the gene neighbor methods giving substantially better performance than any phylogenetic profile method for most organisms; and (4) a study of the effect that the training data has on the performance of the combination methods for the genome context scores, resulting in the very important conclusion that cross-validation results commonly presented in the literature can be overly optimistic about the benefits that can be achieved from combination.

Direct comparison of results across papers in the area of genome context methods are many times impossible due to changes in databases, testing protocols and definition of performance measures, sometimes leading to apparent contradictory conclusions. This was one motivation for the thorough study presented in this paper. In Section 4, whenever possible, conclusions found in the literature will be contrasted with our own conclusions.

## 2 Genome Context Methods

Given two genes *G*_1 _and *G*_2 _in a certain target genome, we wish to compute a measure of the likelihood that they are functionally related. We study the four types of genome context methods widely used in the literature: phylogenetic profiles, gene neighbor, gene cluster, and gene fusion (or Rosetta Stone). Three of these methods rely on information about the presence of homologous sequences for the genes in the target genome on a list of reference organisms. In genome context methods, homology is generally inferred using the degree of sequence similarity given by the E-value. Even though sequence similarity does not directly imply homology, this is a practical and computationally efficient way to infer it commonly used in bioinformatics. The sequence similarity information used here is obtained from the Comprehensive Microbial Resource (CMR) database [20]. The CMR database is loaded into an Oracle relational database using the BioWarehouse toolkit [21] for ease of access. We consider a sequence to be homologous to the query sequence if the E-value obtained from CMR is smaller than a certain threshold *E*.

In this paper, the value of the threshold *E *is tuned to optimize the performance of the methods. CMR uses blastp to generate the E-values. Matches with percent of similarity smaller than 40% or percent of identity smaller than 10% are discarded. Two filters are used by CMR to mask off segments of the query sequence that have low compositional complexity: SEG [22] and XNU [23].

Genome context methods can be grouped into two categories: *full coverage and restricted coverage*. Full-coverage methods are those that can generate scores for all possible gene pairs from a genome, while restricted-coverage methods generate scores only for some pairs. The phylogenetic profile and the gene neighbor methods are full coverage, while the gene cluster and the gene fusion methods are restricted coverage. The gene cluster method generates a score only when two genes are adjacent in the genome and coded in the same strand, and the gene fusion method generates a score only when the two genes from the target genome are found fused into a single gene in some other genome. Table 1 shows a summary of the genome context methods implemented in this paper.

**Table 1:** Genome context methods implemented in this paper.

Method (Coverage)	Name	Description
Phylogenetic Profiles (Full)	pp-mutual-info	Similarity between the phylogenetic profiles (PPs) of two genes across a list of reference genomes using the mutual information between the two PPs as similarity measure.
	
	pp-pearson	As above, using the Pearson correlation as measure.
	
	pp-jaccard	As above, using the Jaccard coefficient as measure.
	
	pp-pval	As above, using the p-value of the observed PPs given by the hyper-geometric distribution that assumes that the probability of a homolog of gene *G*_1 _appearing in genome *i *is independent and identical to the probability of a homolog of gene *G*_2 _appearing in genome *j*, for all genes and all genomes.
	
	pp-wpval	Same as pp-pval but relaxing the assumption that the probability of a homolog of a target gene appearing in genome *i *is the same for all *i*.
	
	pp-wpval-with-runs	Similar to pp-wpval but using a heuristic to compensate for the assumption of independence of the genomes.

Gene Neighbor (Full)	gn-lnX	Measure of the relative distance between homologs of two genes in a list of reference genomes. The measure is given by the negative logarithm of the product of the relative distances between genes across all genomes that contain homologs for both genes.
	
	gn-pval	P-value measure for the observed value of gn-lnX.
	
	gn-norm-lnX	Normalized version of gn-lnX where its value is divided by the total number of genomes found to contain both homologs.

Gene Fusion (Restricted)	gf	P-value for the observed number of times two genes are found fused into a single gene in the reference genomes.

Gene Cluster (Restricted)	gc	Relative distance in bases between two genes that are adjacent and coded in the same strand in the target genome.

The short name used in the rest of the paper to refer to each method is listed in the second column. The methods are explained in detail in Section 2.

### 2.1 Phylogenetic Profiles Method

The phylogenetic profile (PP) of gene *G *is a binary vector encoding the presence (indicated with a 1) or absence (indicated with a 0) of a homologous sequence of *G *in a list of reference genomes. The length of the PP vector is given by the number of genomes in the list. Given the PPs for two genes, the *product *PP is defined as the vector that contains a 1 only in the positions in which both PPs have a 1 and 0 otherwise. If we think of the PPs for each gene in a target genome as forming a matrix where each row corresponds to a gene, then the columns of this matrix are the **organism profiles**, while the rows are the **gene profiles**. It is assumed that two genes having similar PPs are likely to be functionally related, since evolutionary pressure favors the simultaneous preservation or elimination of two genes that function together. Several measures have been proposed in the literature to quantify the distance between two PPs. Here, we compare five of the most common measures: mutual information (which, in plots and tables we will call **pp-mutual-info**), Pearson coefficient (**pp-pearson**), Jaccard coefficient (**pp-jaccard**), hypergeometric p-value (**pp-pval**), and weighted hypergeometric p-value (**pp-wpval**). Furthermore, we implement a sixth PP method called weighted hypergeometric p-value with runs (**pp-wpval-with-runs**) proposed in [19] aimed at relaxing some of the assumptions made in the computation of the other metrics. In the following we give mathematical definitions of the first four measures and conceptually describe the remaining two. Given two PPs, *p*_1 _and *p*_2_, for genes *G*_1 _and *G*_2_, where *p_k _*(*i*) is 1 if organism *i *in the reference list contains a homolog of gene *G_k _*and 0 otherwise, define

$$\begin{array}{l} \;\;\;\;\;\;\;n_{k}(x)\;=\sum_{i=1}^{M}I(p_{k}(i)=x),\\ n_{12}(x,\;y)=\sum_{i=1}^{M}I(p_{1}(i)=x,p_{2}(i)=y), \end{array}$$

where *I *is the indicator function, which takes a value of 1 if all the arguments are true, and 0 otherwise, and *x *and *y *take values 0 or 1. The index *i *runs over the *M *organisms in the list of reference organisms. For example, *n*_1_(1) is the number of 1 s in the PP vector for gene *G*_1_. Similarly, *n*_12_(1, 1) is the number of common 1 s in the two PPs, or equivalently, the number of 1 s in the product PP. The four simplest PP measures we use here are defined as

$$\begin{array}{l} {\text{jacc}}_{1,2}=\frac{n_{12}(1,1)}{M-n_{12}(0,0)}\text{,}\\ \text{pva}1_{1,2}=\sum_{i=n_{12}(1,1)}^{M}\frac{\left(\begin{array}{c} n_{1}(1)\\ i \end{array}\right)\left(\begin{array}{c} M-n_{1}(1)\\ n_{2}-(1)-i \end{array}\right)}{\left(\begin{array}{c} M\\ n_{2}(1) \end{array}\right)},\\ {\text{pear}}_{1,2}=\frac{\sum_{i=1}^{M}\left(p_{1}(i)-{\bar{p}}_{1})(p_{2}(i)-{\bar{p}}_{2}\right)}{\sqrt{\sum_{i=1}^{M}{\left(p_{1}(i)-{\bar{p}}_{1}\right)}^{2}\sum_{i--1}^{M}{\left(p_{2}(i)-{\bar{p}}_{2}\right)}^{2}}},\\ {\text{muti}}_{1,2}=\sum_{i,j=0,1},\frac{n_{12}(i,j)}{M}\log\frac{n_{12}(i,j)M}{n_{1}(i)n_{2}(j)}, \end{array}$$

where jacc, pval, pear, and muti are short for pp-jaccard, pp-pval, pp-pearson, and pp-mutual-info respectively, and ${\bar{p}}_{k}=\sum_{i=1}^{M}p_{k}(i)/M$. These PP distance measures assume more or less explicitly that the event "genome *i *contains a homolog of gene *G*_1_" is independent and identically distributed to the event "genome *j *contains a homolog of gene *G*_2_" for any *i *and *j *and any pair of genes *G*_1 _and *G*_2_. That is, they assume that the presence of a certain gene in a certain genome does not influence the presence of any other gene in that genome or in any other genome. This is, of course, not the case when phylogenetically related genomes are present in the reference list, since the existence of a gene in one of the genomes would strongly suggest its presence in a related genome.

The weighted p-value (which we will call **pp-wpval**), relaxes the assumption of the probabilities for "genome *i *contains gene *G*" being identical across all genomes, preserving the assumption of independence. In this method, a p-value is calculated assuming that the probability that a gene *G *from the target genome is contained in genome *i *is given by the fraction of all the genes in the target genome that are contained in genome *i*. There is no closed form expression for the p-value computed under these relaxed assumptions. See [24] for a recursive method to estimate this probability.

In [19], Cokus et al. propose a heuristic to compensate for the effect of making the independence assumption. A tree is created by hierarchically clustering the organism profiles. For this, the Jaccard distance is used, since it is a real distance metric, satisfying the triangle inequality. This tree is then *swiveled *by rotating the left and right branches under each node such that the sum of the distances between neighboring leaves is minimized. By reading the tree leaves in order, a sorted list of organisms is obtained. Cokus et al. then assume that, if PP vectors are obtained from such a list, a product PP vector that contains few runs of consecutive 1 s can be considered as less evidence for functional relatedness than a vector that contains many runs of consecutive 1 s, since consecutive 1 s correspond to phylogenetically related organisms. A heuristic measure designed to capture this intuition is proposed as the ratio between the weighted hypergeometric p-value and a p-value for the observed number of runs in the product PP. We call this measure the **pp-wpval-with-runs**. Cokus et al. compare their heuristic method with a highly sophisticated method proposed earlier with the same goal of relaxing the independence assumption [25]. They find that their method outperforms the more complex and much more computationally expensive method in a random subset of samples used for comparison.

### 2.2 Gene Neighbor Method

The second set of genome context methods studied in this paper incorporates information that the PP methods ignore: the distance between the homologs of two genes on the reference genomes for which both genes have homologs. We implement the gene neighbor method as introduced in [4]. The assumption behind this method is that genes that are located near each other across a set of reference genomes are likely to be functionally related [26].

Consider two genes from the target genome, *G*_1 _and *G*_2_. Let *d_i _*be the distance between the homologs of these two genes in a genome *i *with *N_i _*genes. The distance is computed as the number of genes that appear between the two homologs plus 1. Hence, adjacent genes have a distance of 1. When the two genes *G*_1 _and *G*_2 _have the same homolog in genome *i*, the distance is defined to be 1 instead of 0. This is done to avoid having *x *in Equation (2) below be zero whenever two genes have the same homolog in some genome, which would force us to effectively ignore the distances observed in all other genomes. If the genes are independent, then, for a circular chromosome, the distance (measured in units of genes) between the genes seen as a random variable, *D_i _*, is uniformly distributed between 1 and (*N_i _*- 1)/2 (ignoring boundary details about whether *N_i _*is even or odd and the few cases in which the two homologs are the same gene). Then, the probability that the distance between the two genes is smaller than the observed distance, *d_i _*, is given by

(1) $$p_{i}=\text{Pr}(D_{i}\leq d_{i})=\frac{2d_{i}}{N_{i}-1}.$$

We have defined *p_i _*as the cumulative distribution function of the random variable *D_i _*, but it can also be seen as a random variable *P_i _*= 2*D_i _*/(*N_i _*- 1) measuring the relative distance between the two genes. As a random variable, *P_i _*has a uniform distribution between 0 and 1.

Given $\tilde{M}$ reference genomes in which both genes, *G*_1 _and *G*_2_, have homologous sequences, and assuming independence across genomes, we can compute the joint probability that the distances are smaller than the observed distances, as

(2) $$x=\text{Pr}(D_{1}\leq d_{1},\;\ldots ,\;D_{\tilde{M}}\leq d_{\tilde{M}})=\prod_{i=1}^{\tilde{M}}p_{i}.$$

Our simplest score for the gene neighbor family of methods is given by $-\log(x)=-\sum_{i=1}^{\tilde{M}}\log(p_{i})$. We call this score **gn-lnX**. We also use a normalized version of this score given by log(*x*)/$\tilde{M}$ that we call **gn-norm-lnX**.

As in the case of *p_i_*, *x *is a probability but it can also be seen as a random variable, *X*. Bowers et al. [4] derive a p-value for this random variable. Since *P_i _*has a uniform distribution between 0 and 1, - log(*P_i_*) has an exponential distribution with rate parameter 1 and, hence, $-\log(X)=\sum_{i=1}^{\tilde{M}}-\log(P_{i})$ is the sum of $\tilde{M}$ independent random variables having exponential distributions, and it is distributed as Gamma with scale parameter equal to 1 and shape parameter equal to $\tilde{M}$. Applying the Gamma cumulative distribution function, we get

(3) P(X≤x)=P(−log(X)≥−log(x)) =∑k=0M˜−1e−(−log(x))(−log(x))k/k!=x∑k=0M˜−1(−log(x))k/k!

This formula gives the probability that the product *X *of the relative distances between the homologous sequences of *G*_1 _and *G*_2 _across all genomes where both genes have homologous sequences is smaller than the observed value, assuming that the positions of these sequences are independent of each other and that the distances are independent across genomes. We call this measure, *P*(*X *≤ *x*), the gene neighbor p-value **gn-pval**. Small values of this probability suggest that one or both of these assumptions do not hold. Since what we want to quantify with this measure is the degree of dependence between the locations of the genes, we should choose the reference genomes such that the second part of the assumption holds as much as possible, ensuring that small values of this probability can only be due to the dependence we wish to measure. If highly related genomes are present in the reference list, the second assumption is violated since it is likely that the distances between homologs will be similar across all related genomes and not independent as it is assumed in the computation of the p-value. As we will see, this intuition is supported by our results, where we find that having a list of reference genomes that does not contain highly related organisms is beneficial to the performance of this method.

### 2.3 Gene Cluster Method

Genes that are located on the same operon are more likely to be functionally related than genes that are not. The gene cluster method [4] computes the distance in bases between two adjacent genes that are transcribed in the same direction. This distance has been found to be a good predictor of whether the genes are in the same operon [27]. Instead of using the p-value expression introduced in [4], we simply use the raw distance in bases between the genes multiplied by a factor *m *that aims to normalize this distance to make it genome independent and is given by the number of genes in the genome divided by the total number of intergenic bases in the genome. Since the expression in [4] is monotonically increasing with this normalized distance, both scores lead to identical performance. In plots and figures, we call this measure **gc**.

This method, in contrast to the two previous methods, has restricted coverage. That is, it does not generate a score for every possible pair of genes. In fact, if the target genome contains *N *genes, from the *N*^2 ^possible gene pairs, at most *N *will have a gene cluster score. For the purpose of comparing performance across methods, we assign a plus-infinity score to all gene pairs that do not get a valid gene cluster method. That is, genes that are not adjacent and transcribed in the same direction are considered to be infinitely distant under this method.

### 2.4 Gene Fusion

The gene fusion method [2-4] is based on the assumption that two genes in a target genome that have been fused together in some reference genome, have a high likelihood of being functionally related. Given two genes *G*_1 _and *G*_2 _from the target genome, we search the reference genomes for genes *G_R _*that appear to be formed by the fusion of *G*_1 _and *G*_2_. These genes have been called *Rosetta Stones *in the literature [2]. To declare a gene *G_R _*in the reference genomes to be a Rosetta Stone, we require that *Q% *of the *G*_1 _and *G*_2 _sequences is found in *G_R _*and that the BLAST E-values for both matches are at most *E*. Results for different values of *Q *and *E *are presented in this paper. As a score, instead of simply assigning a 1 when a Rosetta Stone is found and 0 otherwise, we use the p-value given by the hypergeometric distribution as described in [4]. This score lowers the rank of cases in which Rosetta Stones could have been found by chance due to the query genes, *G*_1 _and *G*_2_, being composed of common domains that generate many matches in the reference genomes.

As in the case of the gene cluster method, the gene fusion method has restricted coverage, since it generates scores only for those gene pairs for which a Rosetta Stone has been found in the reference genomes. As for the gene cluster method, when no score is generated by this method for a certain gene pair, an extreme value is assigned, in this case, a p-value of 1.

## 3 Score Normalization

To find which genes in a certain target genome are functionally related, we can compute the genome context scores for all possible gene pairs in the genome. For each genome context method, we can then sort the obtained scores and choose the top *N *scores as positive examples. That is, a threshold is chosen and a decision is made that all pairs for which the score is larger than that threshold are functionally related genes.

**Figure 1:**
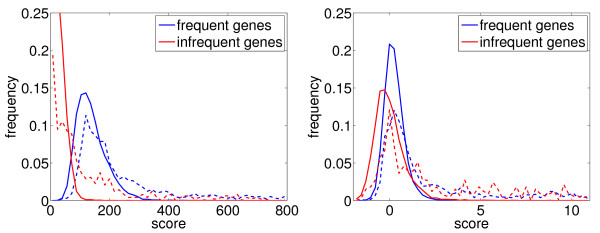
**Distribution of the gn-lnX score (see Table 1) for samples where both genes are frequent and samples where both genes are infrequent, for each sample class**. Dashed lines correspond to positive samples (gene pairs where the products of the two genes belong to the same metabolic or signaling pathway or protein complex), solid lines correspond to negative samples (all other pairs). Note that the curves are distributions, not histograms. Hence, the large unbalance between positive and negative samples cannot be seen here. (Left) Distributions before normalization. (Right) Distributions after znorm composed normalization.

The problem with this procedure is that the scores across different genes might not be comparable with each other. For example, a pair of genes that both have homologous sequences in most organisms in the reference organism list will be likely to have a small value of *x *(Equation 2), simply because many numbers smaller than 1 would be multiplied to obtain *x*. This introduces a bias that makes the gn-lnX scores from different genes incomparable. The threshold chosen to make the final decision will then have to accommodate the biases from the different genes, leading to suboptimal performance. The left plot in Figure 1 shows the distributions of negative and positive samples for the gn-lnX scores, dividing the samples into those where both genes are frequent and where both genes are infrequent. Frequency of a gene is measured as the proportion of organisms in which the gene has a homologous sequence. We can see that samples corresponding to two infrequent genes have a very different distribution of scores than those samples corresponding to two frequent genes. As a consequence, choosing a single threshold to classify samples into positive and negative for both types of samples will lead to clearly suboptimal results. This problem can be seen as an issue of the particular score, and measures can be taken to mitigate the bias. The gn-norm-lnX is, in fact, designed as a bias-compensated version of gn-lnX, since it is normalized by the number of common organisms. Nevertheless, as we will see in the results, data-driven techniques for normalizing the scores can lead to better results than trying to guess the source of the bias and compensating for it using some heuristic.

The problem of score bias occurs in several types of data. Within bioinformatics, the most common example of data that suffers from this problem is microarray data. Several types of normalization methods have been proposed in the literature to compensate for the bias in this type of data. In this paper we extend two of these normalization methods, which have been shown to perform relatively well compared to other methods [9,28], to the genome context scores. The idea behind both methods is to force the distribution of scores to be equal across all *elements*. In the case of the microarray data, the elements are the individual arrays. In our case, the elements are genes within the target genome.

We can arrange the genome context scores for a certain target genome in a matrix *M*. Each entry in this matrix is the score value for a pair of genes. Since all genome context methods are symmetric on the two genes in the pair, *M *will be a symmetric matrix. We can now apply a transformation to each row to equalize the distribution of all rows. We explore two different transforms: z-normalization (**znorm**) and rank-normalization (**rnorm**), also called *quantile normalization *[28].

Znorm computes the mean *μ_i _*and standard deviation *σ_i _*over each row, and normalizes each score *M_ij _*using the expression

(4) $$M_{ij}^{\text{norm}}=\frac{M_{ij}-\mu_{i}}{\sigma_{i}}.$$

After this transformation is applied, each row will have zero mean and unit standard deviation.

A more sophisticated transformation was proposed for microarray data in [9]. In this case, the empirical distribution of the rows is forced to coincide with a target *mean *distribution. This target distribution is obtained by sorting the elements of each row, creating a new matrix *M_s _*, and then finding the mean over each column. The original scores are then transformed by mapping them to the mean value corresponding to the rank of the score within its row. We also explored the use of target distributions given by the median over each column of *M_s _*and by the rank divided by the total number of columns. This last target distribution corresponds to mapping each row to a uniform distribution. Neither of these two alternatives gave a consistent gain over using the mean distribution and, hence, results are not shown for these cases. For both normalization methods, the resulting normalized matrix is not symmetric. Using such a matrix would result in a different score for gene pair (*G*_1_,*G*_2_) than for (*G*_2_,*G*_1_). We solve this problem by creating a new matrix formed by the average of the normalized matrix and its transpose.

Finally, both procedures described above effectively compensate for the bias introduced by one of the genes in the pair, ignoring the effect from the other gene. After averaging the resulting matrix with its transpose, the effect of both genes is taken into account in the final score, although independently from each other. One simple approach to jointly compensate for the effect of both genes is to flip the matrix obtained by normalizing over the rows along its diagonal and repeat the normalization procedure. This compensates for the bias due to the other gene in the pair, *after *the effect of the first gene has been considered. The resulting matrix is again averaged with its transpose to convert it into a symmetric matrix. We call this procedure a *composed *normalization, and denote it by adding-comp to the name of the basic normalization method.

The right plot in Figure 1 shows the distribution of the same scores as in the left plot after znorm composed normalization was applied to the scores. We can see that scores for frequent and infrequent genes are now much better aligned. A single threshold can now be used to classify into positive and negative samples for both groups of samples.

## 4 Results and Discussion

This section presents a large number of experimental results on the genome context methods described in previous sections. We explore the space of parameters of the different methods, including the reference list of organisms and the normalization methods. We also present results on different target organisms and on the combination of individual methods to generate a single unified genome context score.

Results are presented on 10 target organisms chosen from tier 1 and tier 2 of BioCyc version 13.1 [29-32]. These organisms have gone through a relatively large amount of curation through the years and, hence, we believe that we can determine a gold standard of functional relatedness for these genomes with reasonable accuracy.

The reference genomes used to compute the genome context methods are obtained from BioCyc [29,30] version 13.1. From the 481 organisms available in this version of BioCyc, some organisms for which the gene names in CMR could not be reliably mapped to gene names used in BioCyc were discarded. The full list of reference organisms used in this paper contains 460 organisms. This list is available at http://brg.ai.sri.com/functional-relatedness/.

### 4.1 Gold Standard of Functional Relatedness

Two proteins might be functionally related for a variety of reasons, some of which are ill defined. Proteins might bind with each other in a transient manner or in a stable manner (forming a protein complex). Proteins might take part in the same metabolic or signaling pathway. Proteins could also be considered functionally related if, for example, they are expressed in the cell under the same environmental conditions, and are involved in the cell's response to those conditions.

Ideally, a gold standard for testing genome context methods should label pairs of proteins that are functionally related for *any *reason as positive samples, and everything else as negative samples. Unfortunately, bioinformatics databases do not normally contain manually curated information about all possible types of functional relationships between proteins and, hence, positive samples for genome context methods are usually created using a subset of the possible cases. In many papers, pairs of proteins belonging to the same metabolic pathway are taken to be the positive samples [4,10,12,14]. Note that, in this case, the gold standard will be affected by the way a pathway is defined. In [33], Green and Karp show that a pair of genes randomly selected from a BioCyc pathway [30] is more likely to be related by a genome context method than is a pair of genes randomly selected from a KEGG pathway [34]. Other databases for testing genome context methods include as positive samples proteins that belong to the same protein complex [7,12,35], or that were found to interact in some protein-protein interaction database [12].

In this work, samples are given by pairs of genes instead of pairs of proteins. Since for bacterial genomes the correspondence is mostly one-to-one, this choice has little effect in the gold standard.

We label a pair of genes as a positive sample if the products of the two genes catalyze reactions in the same metabolic pathway, belong to the same protein complex, or take part in the same signaling pathway. With this definition we are including all positive cases available from manual curation in the BioCyc databases (EcoCyc version 13.1 contains 922 protein complexes and 300 pathways curated from the literature. The BioCyc tier 2 databases also contain curated complexes and pathways, some of which were curated from the literature, others of which were inferred by curators). This gold standard fails to label as positive samples pairs of genes whose products bind with each other in a transient way, and, perhaps, other less well-defined cases of functional relatedness.

A simple approach to obtain negative samples for the gold standard would be to take all possible gene pairs in the target genome, find the positive samples from the information available in the database and label all other pairs as negative samples. This approach would work well if all genes in the genome had been curated with their function (or functions). Nevertheless, as discussed above, to this date, no genome has been fully annotated with functions for all its genes and, hence, a gold standard obtained by the procedure above would contain some proportion of negative samples which are actual positive samples yet to be discovered or annotated. To avoid this problem, Hu et al. [15] include as negative samples only gene pairs whose products belong to different pathways that do not overlap [15]. In this paper, we choose a similar, though slightly more inclusive, approach. We define the gold standard to contain only pairs of genes for which some knowledge of their function is available. The resulting set includes all possible positive samples within the full set of gene pairs from a genome, but discards the portion of the negative examples that is more likely to result on labelling errors. A gene is included in the list of known-function genes if it satisfies any of the following conditions: (1) the gene encodes an enzyme that catalyzes a reaction present in our database, (2) the gene has been annotated with a leaf node from the GO molecular function ontology, (3) the gene encodes a protein that is a substrate in a reaction, or (4) the gene encodes a protein that is a sigma factor or a transcription factor of known function. We call this set of samples, including both positive and negative samples, the **known-function **set.

For the experiments focused on parameter tuning, we further reduce the number of pairs considered with respect to the known-function set by choosing genes that are labeled as enzymes that catalyze small-molecule reactions in the annotated genome and, hence, are likely to be members of metabolic pathways. We will call these genes sm-enzyme genes, for short. This reduces the number of total pairs for *Escherichia coli K-12 *by a factor of 10 and allows us to run the large number of experiments we present here. Furthermore, the gold standard for the subset of samples involving only genes that are labeled as enzymes in BioCyc is likely to contain relatively fewer errors than the known-function set, since genes that are known to produce enzymes have usually undergone more study than those that are not, giving us somewhat higher confidence on the gold standard. We call these samples the **sm-enzyme set**. Results in Section 4.7 show that the most important conclusions obtained on the sm-enzyme set do not vary when the known-function set of genes is considered. Combination results in Section 4.8 are presented on both sets of samples.

Table 2 shows the list of target organisms considered with the total number of genes, the number of genes used in the known-function set and the number used in the sm-enzyme set, the total number of samples obtained by pairing up those sm-enzyme genes (given by (*e *- 1)*e*/2, if *e *is the number of sm-enzyme genes), the proportion of positive samples within those and the fraction of genes in the genome that have homologous sequences in *Escherichia coli K-12 *(ECK12). This last measure gives an idea of the similarity between the corresponding organism and ECK12. The rows in this table are sorted by this last column. Hence, organisms closer to the top are more *similar *to ECK12 than those closer to the bottom. The short names in the first column of this table will be used throughout the rest of the paper to identify the organisms. The difference between the known-function genes and the sm-enzyme genes is much larger on ECK12 than in the other organisms. This is a direct consequence of the large amount of manual curation that has been done in this database.

**Table 2:** List of target organisms used in the experiments.

short name	full name	**#gen**.	**#w/fn**.	**#enz**.	**#samp**.	%pos	weight
ECK12	*Escherichia coli K-12 substr. MG1655*	4496	3185	1000	495510	0.621	1.000

EC157	*Escherichia coli O157:H7 EDL933*	5475	1032	706	248865	1.327	0.884

ECCFT	*Escherichia coli CFT073*	5379	721	577	166176	0.968	0.856

SHIG	*Shigella flexneri 2a str. 2457T*	4207	886	597	177310	1.020	0.795

VCHO	*Vibrio cholerae O1 biovar El Tor str. N16961*	3949	863	583	169653	1.244	0.528

CAULO	*Caulobacter crescentus CB15*	3818	919	615	188805	0.964	0.362

MTBC	*Mycobacterium tuberculosis CDC1551*	4235	760	569	161596	1.157	0.238

MTBR	*Mycobacterium tuberculosis H37Rv*	3968	811	578	166753	1.409	0.238

FRANT	*Francisella tularensis tularensis SCHU S4*	1671	491	317	50086	1.512	0.230

HPY	*Helicobacter pylori 26695*	1609	415	228	25878	1.946	0.155

The columns correspond to the short and full names for the organism, the total number of genes in the genome, the number of genes with some functional annotation, the number of genes that encode enzymes that catalyze small-molecule reactions, the total number of pairs of such genes (which corresponds to the number of samples used to report results, except in Sections 4.7 and for some results in Section 4.8 where all known-function genes are paired to create samples instead of just small-molecule enzymes), the proportion of positive samples among these samples, and the fraction of genes in the genome that have homologous sequences in ECK12. Organisms are sorted by this last column.

The gold standard for all organisms in table 2 for the sm-enzyme and the known-function sets are available for download at http://brg.ai.sri.com/functional-relatedness/.

### 4.2 Performance Measures

Genome context methods studied in this paper output continuous numeric values that we call *scores*. Given the output of a certain genome context method for gene pairs in a target organism, a classification decision can be made by choosing a threshold and deciding that anything above that threshold corresponds to a positive sample. Depending on the final goal for obtaining functional relationship labels, one might want to choose a conservative threshold that selects only very high confidence positive samples, or a looser threshold that allows for detection of a larger fraction of all positive samples. In the following discussion, and in the rest of the paper, we will assume that higher values of a score indicate stronger evidence of functional relatedness. If the original scores do not comply with this assumption (as in the case of p-values where values closer to zero indicate stronger evidence of functional relatedness), we simply reverse their sign. Given a certain threshold, the number of true positives (tp), false positives (fp), true negatives (tn), and false negatives (fn) can be obtained. True positives and negatives are samples correctly labeled by the system (that is, positive samples for which the score was larger than the threshold or negative samples for which the score was smaller than the threshold). False positives and false negatives are samples for which the system assigned the wrong label. Several measures of performance based on these numbers can be computed. In this work we will use sensitivity and specificity, which are defined as tp/p and tn/n, where p is the total number of positive samples and n, the total number of negative samples.

Sensitivity and specificity can be computed only after a threshold has been chosen, which implies having made a decision about the cost incurred when committing each type of error. A more general way of evaluating a system, without focusing on a certain application or sets of costs, and without making a decision about the threshold we wish to use, is through ROC curves, which show the sensitivity and specificity values at each possible threshold along the range of the score. A system *S*_1 _that has higher specificity values than another system *S*_2 _for any sensitivity value can be declared better than *S*_2_, independently of the application.

In this work, we will present ROC curves focused on the region of high specificity. This is the only useful region of the ROC curve for this task since, given the highly unbalanced number of samples, where around 99% are negative, low specificity values would result in a very large proportion of false positive samples. For example, a specificity value of 90% would correspond, in the case of ECK12, to 49,642 false positives for the sm-enzyme set. At that level of specificity, the best genome context method gives a sensitivity of around 48%, which corresponds to approximately 1,400 true positives. Hence, at that operating point only around 3% of the selected samples are true positives. Lower values of specificity make the situation even more extreme. This criterion of reporting results on the top scoring pairs is used in most genome context papers when showing the cumulative accuracy in the top *p*% samples as sorted, from largest to smallest, according to their score values. In those cases, the largest value of *p *is chosen to be a small percentage of the total number of gene pairs (up to 25,000 samples are selected for *E. coli *in Figure 5 in [4] and 10,000 in [19], out of around 10 million possible pairs).

Apart from showing ROC curves, we will show curves of sensitivity when the threshold is chosen such that the top *p*% of the samples are labeled as positive, for different (small) values of *p*. This allows us to compare many methods at once in a single figure, which would not be possible using ROC curves.

### 4.3 Results for Individual Genome Context Methods

We present results for each of the genome context methods, comparing their performance for different values of the relevant parameters for the method. Results in this section are presented on the sm-enzyme gold standard set for ECK12 (first line in Table 2). As reference organisms we use the complete set of 460 organisms from BioCyc. For the two scores, pp-wpval and pp-wpval-with-runs, that are computed using iterative algorithms and suffer numerical underflow problems when the reference list is too big in size (or when the weights of the organisms involved are too close to 0 or 1 [19]), we use a subset of 216 organisms obtained by clustering (see Section 4.4) from the full list.

#### 4.3.1 Results for full-coverage methods

As explained in Section 2, the phylogenetic profile, gene neighbor, and gene fusion methods all use homology information for their computation. In this work, as is generally done in implementations of genome context methods, two genes are assumed to be homologous if the sequence similarity E-value returned by BLAST is smaller than a certain threshold. Thresholds from 10^-10 ^[4] to 10^-4 ^[12,17] have been reported in the literature. In the additional file 1, we explore the performance of all genome context methods for a range of E-value thresholds from 10^-10 ^to 10^-3 ^and find 10^-4 ^to be approximately optimal. This is the value we use for the rest of the experiments unless explicitly stated otherwise.

**Figure 2:**
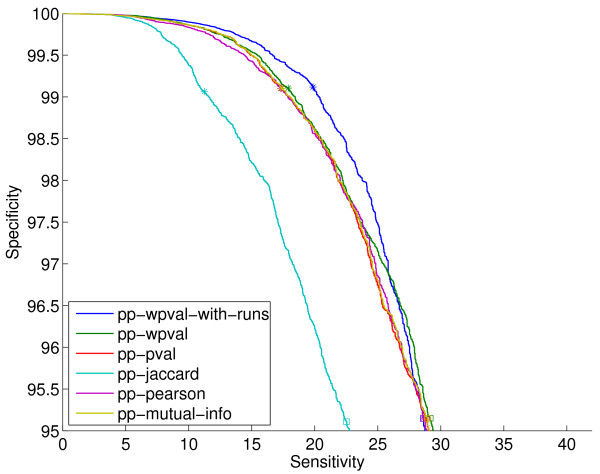
**ROC curves on the sm-enzyme gold standard set for ECK12 for the phylogenetic profile method using different distance metrics and *E *= 10^-4^**. The stars and squares correspond to the sensitivity and specificity obtained for each system when choosing the top 1% and 5% samples. The full list of reference genomes is used to generate all these scores except pp-wpval and pp-wpval-with-runs, for which a smaller list of size 216 obtained by clustering is used.

Several methods can be used to measure the distance between two phylogenetic profiles, as discussed in Section 2.1. Figure 2 shows (the upper-left corner of) the ROC curves for the six different distance measures. We see that, except for the Jaccard coefficient, all other simple measures lead to similar performance. The most sophisticated measure, pp-wpval-with-runs gives a gain over the simpler measures at the operating points considered here (this region is comparable to the region used to present results in the original paper of Cokus et al. [19]), although it is substantially worse than the others at lower specificity points (not shown). The weighted p-value is slightly better than the other simpler measures. Nevertheless, pp-wpval requires an expensive iterative algorithm for its computation. It does not seem that the gain in performance is worth the extra computation cost.

**Figure 3:**
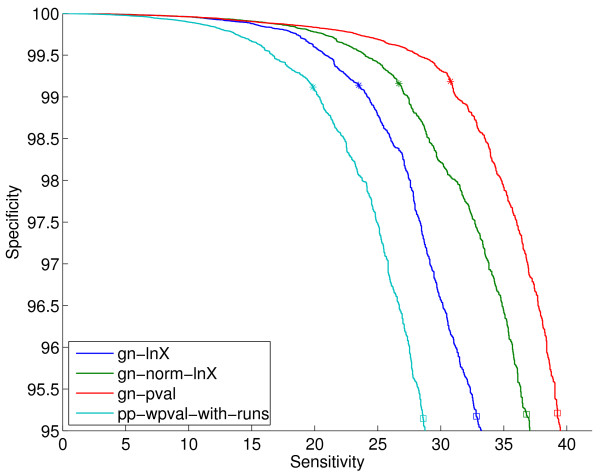
**ROC curves on the sm-enzyme gold standard set for ECK12 for the gene neighbor methods and for the best phylogenetic profile method from Figure 2, pp-wpval-with-runs using *E *= 10^-4^**. See caption for Figure 2 for an explanation of the stars and squares. The full list of reference genomes is used to generate all gene neighbor scores.

Figure 3 shows the ROC curves for the three different gene neighbor scores for *E *= 10^-4^, and, for comparison, the pp-wpval-with-runs scores from Figure 2. The gn methods are clearly better than the pp methods over all operating points of interest. The fact that gene neighbor methods outperform the simpler methods based on only the phylogenetic profiles was also observed in [4], where the gn-pval measure was first introduced. Sun et al. [12] found a different trend, but their comparison is based on final decisions made by each method after thresholds have been chosen. As a consequence, their comparisons are confounded by the fact that methods generate different numbers of positive decisions (with differences of a factor of 20 between some of them). Hence, we believe that their results are not indicative of the relative performance of the methods since arbitrary operating points are chosen for each of them, or forced by the methods themselves, since their implementation of some of the methods does not output scores but hard decisions.

#### 4.3.2 Results for restricted coverage methods

**Figure 4:**
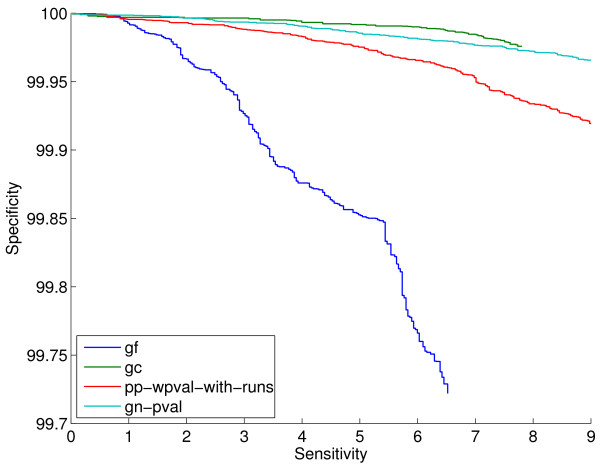
**ROC curves on the sm-enzyme gold standard set for ECK12 for the gene cluster method (gc), the gene fusion method (gf), the best gene neighbor method (gn-pval) and the best phylogenetic profile method (pp-wpval-with-runs)**. *E *= 10^-4 ^and *Q *= 50 are used. The full list of reference genomes is used to generate all scores except pp-wpval-with-runs, for which a smaller list of size 216 obtained by clustering is used.

Additional file 1 shows results when varying the two tunable parameters in the gene fusion method (see Section 2.4): the E-value threshold *E*, and the minimum percent of overlap required between the query genes and the matching gene, *Q*. We find that *Q *= 50 and *E *= 10^-4 ^give a good trade-off between coverage and performance. We use these values for any gene fusion scores in the rest of the paper. Figure 4 shows the ROC curves for the gene cluster method, which has no tunable parameters, the gene fusion method with the parameters indicated above, and the best scores from the gene neighbor and the phylogenetic profile families. Note that this figure shows a much smaller corner of the ROC curve than Figures 2 and 3. We see that the gc method achieves higher specificities than the best full-coverage method (gn-pval), for sensitivities of up to about 8%. Higher sensitivities cannot be achieved by the gc method because of its restricted coverage nature.

### 4.4 Results Varying the Reference Organism List

This set of experiments explores the effect of the choice of reference organisms used to compute the genome context scores. As in Section 4.3, we present results on the sm-enzyme gold standard set for ECK12. The list of reference organisms is then varied by subsetting the complete list of 460 organisms. This study is motivated by the fact that, as we commented in Section 2, most genome context scores rely on an assumption of independence of the genomes in the reference list. When many closely related genomes are present in the list, this assumption is violated, potentially resulting in degradation of performance. To eliminate closely related organisms from the reference list, a clustering procedure is used. Organisms are grouped into closely related clusters and a representative organism is chosen for each cluster, discarding all other organisms in the cluster. The clusters are obtained with the same algorithm used to compute the pp-wpval-with-runs scores: hierarchical clustering using the Jaccard distance on the organism phylogenetic profiles as similarity measure between organisms. Hierarchical clustering creates a tree where the root corresponds to a single cluster containing all organisms, the nodes correspond to smaller and smaller clusters and the leaves correspond to individual organisms. By pruning this tree at different levels, different numbers of clusters can be obtained. A principled way to perform the pruning is to enforce a certain maximum within-cluster distortion. A maximum distortion of 0 would only be satisfied by the leaves. As the maximum distortion is increased, the pruning occurs higher up in the tree and larger and fewer clusters are selected. For each cluster, the representative organism is chosen as the one that has the smallest average distance to all the others in the cluster. The resulting lists of reference organisms are available for download at http://brg.ai.sri.com/functional-relatedness/.

The clustering procedure not only reduces the bias of the reference list toward certain regions of the phylogeny (the desired effect), but also reduces its size (a potentially undesirable effect). To assess the effect that the size of the list has when no special selection of organisms is performed, we also present results when the smaller lists are obtained by random sampling of the original list. Three different random lists are run for each method. The results for random lists presented in the figures correspond to the performance averaged over these three lists.

**Figure 5:**
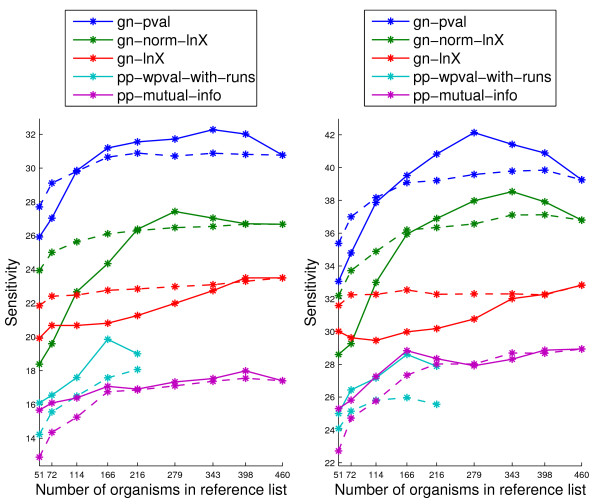
**Sensitivity within the top 1% samples (left) and top 5% samples (right) on the sm-enzyme gold standard set for ECK12 as sorted by each genome context method, varying the list of organisms used for computing these scores**. Parameter *E *is set to 10^-4^. Solid lines correspond to subsets of organisms chosen by the clustering method, and dashed lines correspond to a random selection of organism subsets. The full reference list corresponds to the right most point in each figure (number of organisms equal to 460).

Figure 5 shows the sensitivity within the top 1% and 5% samples as sorted by each genome context method, varying the list of organisms used for computing these scores and the method used to obtain the subsets. The genome context scores shown in this figure are a selected subset of those we implemented, including all three gene neighbor methods and two phylogenetic profile methods. From the pp methods, we keep the best method on the operating points of interest, pp-wpval-with-runs, and one of the simpler ones, pp-mutual-info. For the pp-wpval-with-runs score we report results only for smaller list sizes since, as mentioned earlier, the computational and algorithmic issues become much more complex when the size of the list becomes large. As described before, the size of the lists (x-axis in the figure) is varied by changing a threshold in the clustering algorithm that determines the maximum within-cluster distance. The size of the random lists is fixed to coincide with the sizes of the lists obtained by clustering.

We can see that, for small lists, the random method results in better performance. We believe that this is the case because when few organisms are chosen with the clustering method, a large proportion of the chosen organisms do not have enough homologous sequences of the target organism's genes. For example, when 51 organisms are chosen by clustering, only 17 of them have homologous sequences for more than 20% of the ECK12 genes. On the other hand, when 51 organisms are chosen at random, 38 of them have homologous sequences for more than 20% of the ECK12 genes.

The most interesting conclusion from Figure 5 is that, if the number of clusters is chosen correctly, clustering gives an advantage over using the original list of organisms. In particular, the best genome context method, gn-pval, improves around 7% on the top 5% samples when choosing the list of organisms by clustering relative to the result obtained using the full list of organisms. This same tendency was reported in [17] for the phylogenetic profile method when using a mutual information distance metric. We believe the reason for the gain obtained from the smart selection of organisms is that the computation of the genome context scores assumes independence of the organisms in the list. This, of course, is far from true in the full list of reference organisms, which contains several large groups of related genomes. For example, the list contains 9 strains of *Escherichia coli *and 11 of *Streptococcus pyogenes*. After clustering, on the other hand, fewer correlated organisms appear in the list and the assumption of independence is satisfied more closely. For example, lists of size 279 or smaller contain a single strain of *Escherichia coli *and a single strain of *Streptococcus pyogenes*.

The fact that going beyond 300 organisms seems to be unnecessary for most genome context methods is a direct consequence of the size and composition of our original list of 460 organisms. If a list of *M *organisms widely spread across the phylogenetic tree was available, then perhaps the optimal list size would be closer to *M*.

The gn-lnX score seems to be an exception to most of the observations made above. As mentioned earlier, this score is highly affected by bias across different genes. As we will see, after normalization, this score behaves very similar to gn-pval.

The best sensitivity in these curves is around 32% for the top 1% samples. That is, when choosing the top 1% samples, we find around 32% of all the functionally related gene pairs in the database. For ECK12, a score that has 32% sensitivity corresponds to a proportion of positive samples among the top 1% samples of around 24%. That is, around 76% of the selected samples are errors. This apparently low accuracy is due to the enormous imbalance between the two classes of samples. As Table 2 shows, less than 0.62% of the gene pairs in ECK12 are positive samples. A system that randomly labeled a sample as positive would then have 0.62% accuracy. A 24% accuracy is then a **38-fold improvement **with respect to making a random decision about the functional relationship of the genes when choosing 1% of the total number of gene pairs. Results for the two restricted-coverage scores, gf and gc, are not presented here. For gc, the reference organisms list is not used and, hence, its performance does not vary with the chosen list. For gf, we found that the larger lists of size above 300 lead to very similar performance and coverage. Smaller lists do not degrade the performance, but restrict the coverage. Overall, the list of size 343 is approximately optimal across methods from the four families. This is the list size we use for the rest of the experiments described in this paper unless explicitly stated otherwise.

### 4.5 Normalization Results

**Figure 6:**
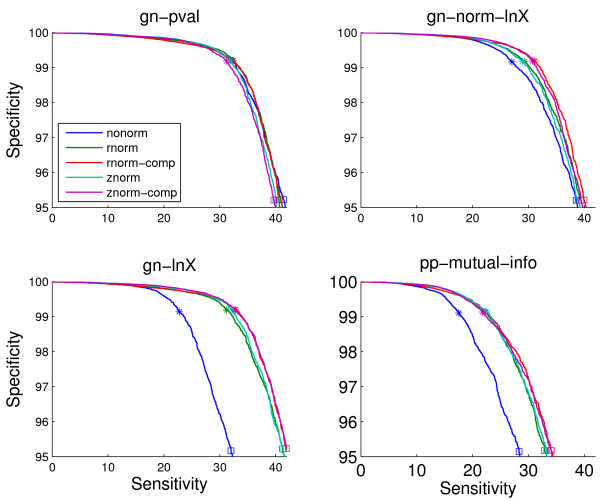
**ROC curves on the sm-enzyme gold standard set for ECK12 for four genome context methods using different types of normalization procedures: znorm, rnorm and their composed variants, and the original unnormalized scores (nonorm)**. Parameter *E *= 10^-4 ^and a reference list of size 343 obtained by clustering are used to generate these scores.

Figure 6 shows the results for four genome context scores for different kinds of normalization methods when using the organism list of size 343 obtained by clustering. Conclusions about normalization performance do not vary widely across lists. Results for all lists will be presented later in this section for a selected normalization procedure. Normalization results are shown only for the full coverage genome context methods. For the other methods, normalization can be performed by ignoring the entries in the matrix corresponding to gene pairs for which no valid score was produced by the method when estimating the statistics for the row. Nevertheless, for these scores normalization does not lead to improvements, and it is, arguably, not reasonable to expect so since only a small percentage of the pairs involving a certain gene generate a valid score with which to estimate the statistics.

We note that, even though results are presented on a subset of all possible gene pairs from the target genome (in this case corresponding to sm-enzymes), the normalization is performed on the full matrix of scores where the columns and rows are all the genes in the target genome. This is the only fair way to perform the normalization since it does not assume any knowledge about the function of the genes. Furthermore, fortunately, we have observed that using the full matrix of scores to perform normalization is, in fact, better than using the matrix of scores corresponding to the subset of pairs being used to report results.

We see that large improvements are obtained from normalization from two of the four genome context scores shown in Figure 6, gn-lnX and pp-mutual-info, and more modest improvements for a third one, gn-norm-lnX. After normalization, gn-lnX outperforms gn-norm-lnX, which was substantially better before normalization. These results demonstrate the advantage of compensating for the bias in a data-driven manner instead of through the use of heuristics. Results for the other pp methods are similar to those for pp-mutual-info, showing large improvements after normalization. The exception to this is pp-wpval-with-runs, for which smaller gains from normalization are observed compared to the other PP methods.

We believe the lack of gain from normalization on the gn-pval method is due to a combination of two effects. First, gn-pval is intrinsically more immune to gene-dependent biases than gn-lnX given that it is already normalized by definition for one of the biggest sources of bias in gn-lnX: the number of organisms that contain both genes. This same reason also explains why gn-norm-lnX shows little gain from normalization. Second, gn-pval has a very different distribution from gn-lnX, having very marked peaks at 0 and 1, probably making the estimation of a normalization transformation less robust.

The composed versions of the two normalization methods lead to modest gains with respect to the simpler versions. Since the gains are small and, as we discuss later, these gains do not hold for the known-function gold standard set, we choose to use the non-composed version of znorm for the rest of the paper. We choose znorm over rnorm for its simplicity, since both lead to very similar performance.

**Figure 7:**
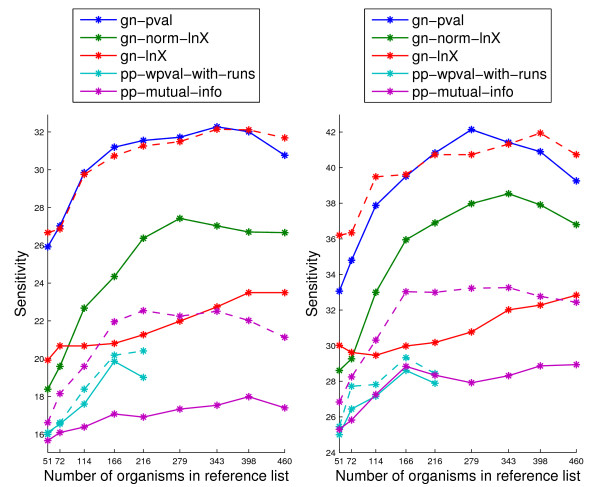
**Sensitivity within the top 1% samples (left) and top 5% samples (right) on the sm-enzyme gold standard set for ECK12 varying the list of organisms used for computing these scores for the unnormalized scores (solid lines) and the znorm scores (dashed lines)**. Solid lines coincide with the solid lines in Figure 5. Normalization results are only shown for the three genome context methods for which it gives improvements to reduce clutter. Parameter *E *is set to 10^-4^.

Figure 7 shows the sensitivity for the top 1% and 5% samples for the unnormalized scores and for the znormed scores for reference lists of different sizes with the organisms chosen by clustering. Normalization results are shown only for the three scores for which it gives a significant gain to avoid clutter. For these methods normalization helps across all lists. After normalization, gn-lnX becomes comparable to gn-pval (unnormalized). From the pp methods, pp-mutual-info greatly benefits from normalization, resulting in better performing scores than pp-wpval-with-runs, which for the top 1% samples was substantially better before normalization.

**Figure 8:**
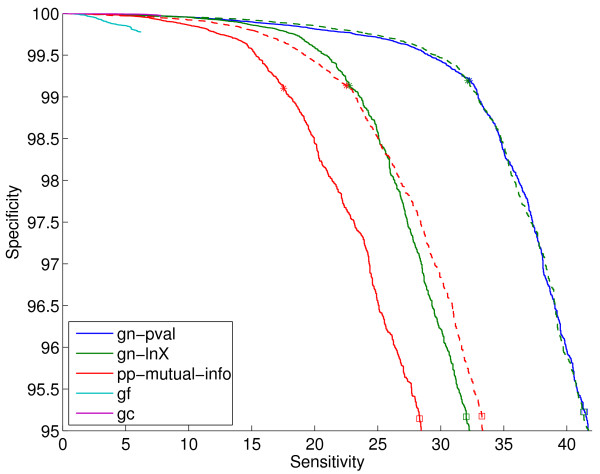
**ROC curves on the sm-enzyme gold standard set for ECK12 for selected genome context methods: the best two gene neighbor methods, gn-pval and gn-lnX.znorm, and its unnormalized version gn-lnX, the best phylogenetic profile method after normalization, pp-mutual-info.znorm, and its unnormalized version, pp-mutual-info, the gene-fusion method, gf, and the gene cluster method, gc**. Normalized version are shown in dashed lines with the same color as the corresponding unnormalized method. Parameters *E *= 10^-4 ^and *Q *= 50 and a reference list of size 343 obtained by clustering are used to generate these scores.

Finally, Figure 8 shows the ROC curves for selected genome context methods for the list size of 343. We can see that, as we already observed in Figure 7, gn-pval and gn-lnX.znorm are almost indistinguishable at the operating points of interest. Clearly, even after normalization, pp scores are still substantially worse than gn scores. As we have already seen in Figure 4, gene fusion is by far the worst of all methods. Overall, the ranking of the different methods agrees with the findings in Figure 5 in [4].

### 4.6 Results Varying the Target Organism

**Figure 9:**
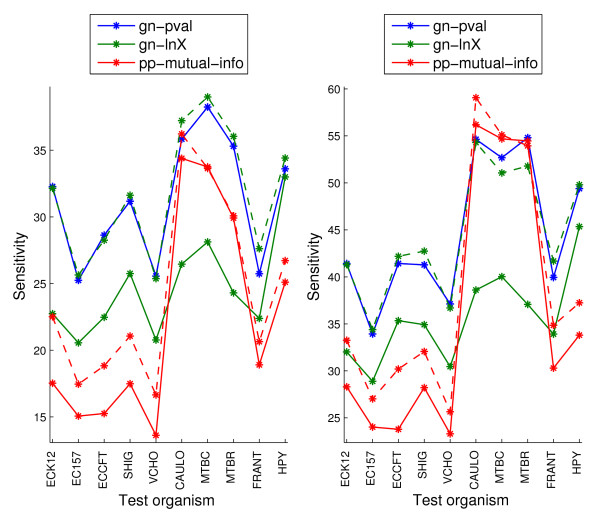
**Sensitivity within the top 1% samples (left) and top 5% samples (right) for three full-coverage genome context methods, on the sm-enzyme gold standard set for different organisms**. Solid lines correspond to the unnormalized scores and dashed lines correspond to znormed scores. Parameter *E *= 10^-4 ^and a reference list of size 343 obtained by clustering are used to generate these scores.

Figure 9 shows the results for the different target organisms on the sm-enzyme set for three genome context scores (chosen among the full-coverage scores for being the best in their category either before or after normalization) when using the organism list of size 343 obtained by clustering. Solid lines show performance of the original scores, and dashed lines show performance of the znorm scores for the system of the corresponding color. These lines are shown only for methods where normalization gives a gain in performance for ECK12, to reduce clutter. Organisms are sorted as in Table 2. We can see that the ranking of performance of genome context scores is mostly independent of the target organism, with gn-pval and gn-lnX.znorm performing mostly identically to each other and outperforming all other methods across all organisms, except for some cases where the phylogenetic profile methods shows slightly better performance. On all 10 organisms znorm gives gains in either the top 1% or 5% samples over nonorm for the pp-mutual-info score. For the gn-lnX scores, znorm gives gains from 15% to 40% in sensitivity for the top 1% samples, except for HPY, for which only a small gain is observed.

**Figure 10:**
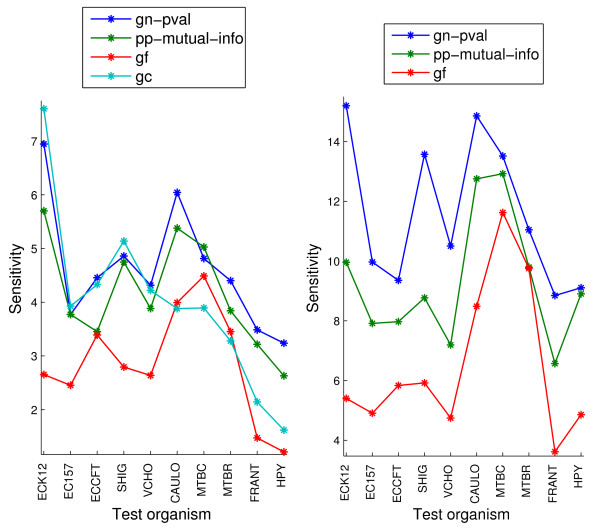
**Sensitivity within the top 0.07% samples (left) and top 0.2% samples (right) for the two restricted-coverage methods, gc and gf, on the sm-enzyme gold standard set for different organisms**. For reference, the performance of gn-pval and pp-mutual-info methods are also shown. Parameters *E *= 10^-4^, *Q *= 50 and a reference list of size 343 obtained by clustering are used to generate these scores.

The restricted coverage scores, gf and gc, generate valid scores for fewer than 1% of gene pairs. The coverage varies across organisms from 0.07% to 0.23% for the gc method and from 0.20% to 0.88% for the gf method. Figure 10 shows the sensitivity within the top 0.2% and 0.07% samples for these two scores and for the gn-pval scores for comparison. Note that, naturally, in this figure, the y-axis corresponds to much lower sensitivity values than in Figure 9, since we are selecting much fewer samples. The general observation from these plots is that the gf method is clearly worse than the gn-pval method for all organisms. The gc method, on the other hand, slightly outperforms the gn-pval method for some organisms. In both Figures 9 and 10, we observe a large variation in the absolute value of the sensitivity across organisms for each genome context method. This behavior might be due to actual inherent differences between the organisms, but it might also be due to varying degrees of curation across them, which makes the gold standard for each organism more or less reliable.

### 4.7 Results Including All Known-Function Genes

All results in the earlier sections were obtained on a subset of all possible gene pairs in the target genomes. The subset corresponds to pairs of genes where both genes are labeled as enzymes that catalyze small-molecule reactions. This was done mainly for computational reasons, to allow for faster execution of the large number of experiments we ran. Furthermore, as mentioned before, we believe that the gold standard for the sm-enzyme subset is likely to be more accurate than for other sets of genes. In this section, we show a subset of results when testing on a larger set of pairs including all genes of known function for comparison. Both gold standard sets are described in detail in Section 4.1.

For ECK12, the sm-enzyme gold standard set contains 10 times fewer samples than the known-function set. This difference is mainly due to negative samples. While the sm-enzyme set contains around half a million samples, of which 3056 are positive, the known-function set contains 5 million samples with 6330 of them being positive. Hence, the ratio of positive-to-negative samples is reduced by 5 times in the known-function set. The positive samples in the known-function set that are not found in the sm-enzyme set correspond mainly to protein complexes. In addition, around 300 of those extra positive samples correspond to pairs of genes whose products are found in macro-molecule reactions and, hence, were not considered in the sm-enzyme set. The consequence of the large difference in the number of negative samples is that, at the same level of specificity, the known-function set contains 10 times more false positives than the sm-enzyme set. Here, we present results in a corner of the ROC curve, corresponding, as we did for the enzyme subset, to choosing around 25,000 samples (as in [4]). This corresponds, for this set of samples, to a minimum specificity of 99.5%.

**Figure 11:**
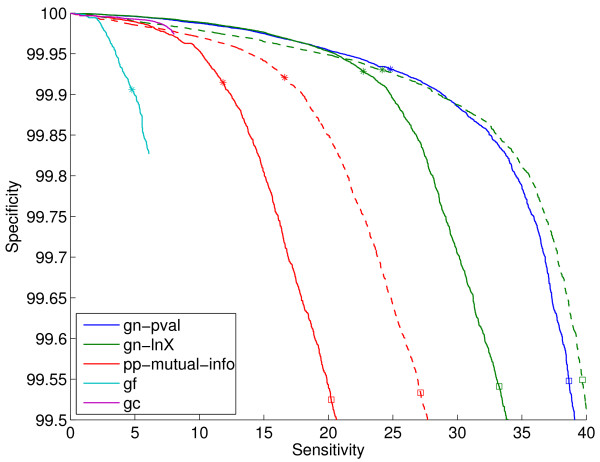
**ROC curves for selected genome context methods on the known-function gold standard set for ECK12 (all previous figures showed results for the sm-enzyme set)**. Parameters *E *= 10^-4^, *Q *= 50 and a reference list of size 343 obtained by clustering are used to generate these scores. This figure is the same as Figure 8, changing only the set of gene pairs on which the methods are tested. Dashed lines correspond to normalized versions of the corresponding scores of the same-color line. The stars and squares correspond to the sensitivity and specificity obtained for each system when choosing the top 0.1% and 0.5% samples.

Figure 11 shows that the qualitative conclusions obtained from the sm-enzyme set are still valid for the known-function set of samples, although the actual details of the relative performance change from one subset to the other (compare with Figure 8). In summary: (1) the gene neighbor methods are substantially better than the phylogenetic profile methods; (2) the gene fusion method is substantially worse than the others; and (3) normalization results in a large improvement in the performance of both gn-lnX and pp-mutual-info. The main qualitative difference between Figures 8 and 11 is that the gene cluster method is now not the best of all methods in the low-sensitivity operating points. Furthermore, even though we do not present results for this observation, we note that the composed normalization methods which, for the sm-enzyme set were giving slightly better performance than the simple methods, do not lead to improved performances in this set. This behavior is likely due to a small set of a few dozen genes that contain very few homologs in the reference lists. The composed normalization procedures do not work well for these genes, resulting in a large number of false positives being generated for them. This is an issue that we plan to address in future research.

### 4.8 Results from Combining Genome Context Methods

Individual genome context methods capture information about the coevolution (or lack of coevolution) of a certain pair of genes in different ways. Each family of methods relies on a different assumption and is based on different information found in the target and reference genomes about the genes in the target genome. Hence, it is reasonable to think that the combination of the individual genome context methods should lead to better performance than any individual method by itself. Here, we explore this issue by implementing combination procedures that take the individual scores from the different genome context methods and fuse them into a single, possibly better-performing, score. We present two sets of combination results using (1) a simple procedure similar to those used in [4] and [14], and (2) decision trees trained using the minimum-message-length criterion. While individual genome context scores do not require the use of a data set with gold standard labels for their generation, most combination methods do require such data set to obtain the optimal combination parameters. In this section we explore the effect that training data has on the performance of the combination for the two combiners under study.

The Prolinks paper [4] and the STRING database [14] use similar approaches for combination. In [4] individual genome context scores are transformed into *confidence measures *by mapping each score value to the percent of positive samples that have a score larger than this value. This measure is called cumulative accuracy in [4]. Cumulative accuracy is computed on the test data itself and cannot be generalized to unseen scores since the mapping is obtained on the particular score values observed by sorting the test scores from larger to smaller and recording the percent of positive samples from top to bottom. The combination is then performed by taking the maximum confidence measure for each sample across all genome context methods. The paper shows that the combination is always better than the best individual method although by a very small amount (see Figures 2 and 5 in [4]). A variation of this method is used in the STRING database [14,16]. In this case, a curve given by hill equations is fitted to the observed cumulative accuracy on the test samples for each genome context method. The scores are then transformed using this curve instead of directly using the cumulative accuracy. After transformation, the scores are combined using the function s = 1 - **∏*_i_***(1 - *s_i_*) where the *s_i_*'s are the transformed scores corresponding to the individual methods [16]. As in the Prolinks case, [14] shows that the combined score is always better than the individual scores, although only slightly.

In this paper, we implement a method similar to that used in the STRING database. The transformation is given by fitting a curve to the cumulative accuracy obtained on a training set, although instead of using hill curves, we fit cubic smoothing splines [36] using the R function smooth.spline [37]. This should not result in any significant difference in conclusions since both types of curves are able to adequately fit the kind of samples obtained from genome context methods. Since the transformation is given by a generic function that can be learned on some data set and applied on another, this method allows us to test the generalization power of the combination approach, which could not be done with the nonparametric transformation used in the Prolinks paper [4]. After the individual scores are transformed, the combination is performed using the product function proposed in [16] and described above. We found this function to work slightly better than the maximum function used in Prolinks in most cases. In the remainder, we call this combiner the *confidence-product combiner*.

The IND software package was used to train the decision tree combiners [38,39]. We compute combination scores using a bagging procedure [40]. The combined score is computed as the average of the output generated by 10 trees trained using different sets of samples. The alternative sets are obtained using the standard procedure of random sampling with replacement from the original set of samples. This *bagging *procedure has been found to lead to significantly better performance than single trees in a wide range of applications. Since the number of negative samples is extremely large and, for ECK12, around 800 times larger than the number of positive samples (on the known-function set), when we choose the set of negative samples used to train each tree, we select only a fraction of them. Hence, if the original training set contains P positive samples and N negative samples, the final training set for each bagged tree will contain P positive samples (sampled *with replacement *from the original P samples, which means that some samples might be chosen twice and some samples might never be chosen) and 10*P negative samples (also chosen by randomly sampling the original set of N negative samples with replacement).

All gene pairs involving a small set of genes (from one to eight genes depending on the organism) for which no homology information is found in the CMR database are ignored when training the trees and, for consistency, when fitting the splines in the simpler combination method. The lack of homology information results in genome context scores that are outliers with respect to the score distribution, which results in strange artifacts on the probabilities output by the trees if those samples are kept during training.

**Figure 12:**
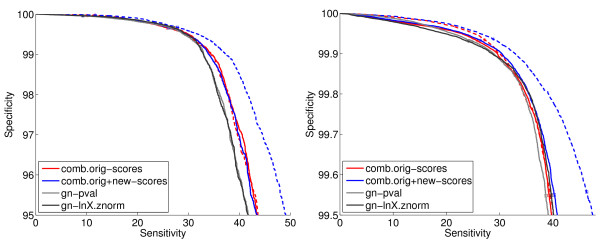
**ROC curves for the sm-enzyme gold standard set (left) and the known function set (right) of samples for ECK12 for different combination strategies**. Solid curves correspond to the confidence-product combiner, and dashed curves correspond to the decision tree combiner. The color of the curve varies according to the scores being combined. Red curves (called **comb.orig-scores**) correspond to combiners using only gn-pval, pp-mutual-info, gc, and gf. The blue curves (called **comb.orig+new-scores**) include those four plus gn-lnX, gn-lnX after znorm and pp-mutual-info after znorm. Parameters *E *= 10^-4^, *Q *= 50 and a reference list of size 343 obtained by clustering are used to generate all scores. Two individual scores are also shown for comparison: gn-lnX after znorm and gn-pval. These are the best two individual systems. A combined system can be considered successful if its curve is consistently better than that of the best individual system being combined at every operating point.

Figure 12 shows combination results when using a 10-fold cross-validation procedure on ECK12 for training the combiners. Normally, cross-validation results are obtained by randomly splitting all available samples (gene-pairs, in our case) into *N *sets and training the combiner for each set on the remaining *N *- 1 sets. Results are then reported on the full set after collecting back the scores obtained on the *N *sets. This is the procedure used, for example, in [7,11]. In this work, we use a modified procedure to ensure that similar pairs are not distributed across sets, since this results in an optimistic estimation of the performance. For this, the ECK12 genes are first grouped into "similar gene" groups. Each group contains all genes that are similar (E-value smaller than 10^-2^) to at least one other gene in the group. Pairs of groups are then randomly split into 10 sets and then expanded to pairs of genes by pairing each gene in one of the groups in each pair with every other gene in the other group of the pair. By using this procedure we ensure that if pair (*G*_1_, *G*_2_) is similar to (*G*_3_, *G*_4_) in the sense that *G*_1 _is similar to *G*_3 _and *G*_2 _is similar to *G*_4_, these two pairs will end up in the same set. The standard cross-validation procedure does not guarantee this and, hence, combiners are trained using pairs that are similar to those in testing. Since similar pairs also have identical or very similar set of genome context scores, the standard cross-validation procedure results in overly optimistic results. In our experiments, the standard cross-validation procedure results in an overestimation of the sensitivity of up to 5% relative to the performance obtained with the proposed cross-validation procedure. Using the proposed cross-validation procedure is particularly important for a fair assessment of the performance when complex combiners, like the decision tree one, are being tested. Two different sets of scores are used for combination in Figure 12. The set called **orig-scores**, corresponds to the four scores standard in the genome context literature: gn-pval, pp-mutual-info, gc and gf. These are the same scores used in [4] and [14]. The set **orig+new-scores **includes those four scores plus some of the scores proposed in this paper: gn-lnX, gn-lnX after znorm and pp-mutual-info after znorm. The color of the curves indicates the set of scores used for combination. Solid curves correspond to the confidence-product combiner and dashed curves to the decision tree combiner. The figure also shows the gn-pval and gn-lnX.znorm curves in gray as a reference. Since these are the best individual scores, combination curves can be compared to these curves to assess the advantage of the procedure over using the single best score. The left plot shows the results for the sm-enzyme set and the right plot shows results for the known-function set. When testing on the sm-enzyme set, the sm-enzyme subset of samples from the training sets is used for training, while when testing on the known-function set, the known-function subset of samples is used for training. We can see that, in both cases, the best combiner is given by the decision tree including the new scores. This is an intuitive result: a relatively more complex combiner is required to make use of all the power of a larger set of scores. The other three combiners are almost undistinguishable from each other and slightly better than the best individual system only for the sm-enzyme set. Overall, we see a gain between 10% and 20% in sensitivity for a fixed value of specificity in the best combined score with respect to the gn-pval score.

The cross-validation results even though fair in the sense that train data is carefully chosen to not contain similar samples to those in testing, can still be considered optimistic. In cross-validation, combiners are trained using gene pairs from the same organism (or set of organisms) than the test gene pairs. As we saw in Figure 9, the performance of the genome context methods varies across different organisms. A combiner trained using samples from one set of organisms might not be optimal in another set. In practice, genome context methods are generally used to generate functional relationship scores for gene pairs that do not belong to an organism for which we have enough annotation to create a gold standard. Hence, to realistically estimate the performance one should expect in practice for a certain combination method, the training data should be obtained from organisms other than those present in the test data.

**Figure 13:**
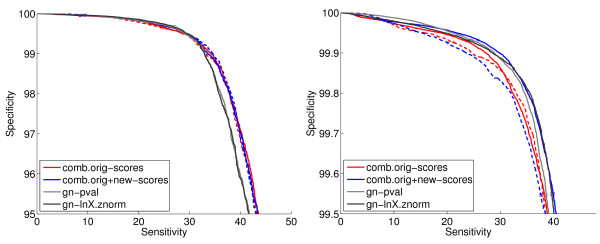
**Same as Figure 12 but training the combiners using samples from CAULO, MTBC, MTBR, FRANT and HPY instead of using cross-validation on ECK12 samples**.

Figure 13 shows the same combination results as in Figure 12 but obtained using training samples from the organisms in Table 2 that are more distant from ECK12: CAULO, MTBC, MTBR, FRANT, HPY. The other organisms in the table are not used for training since they are too similar to ECK12 and would, again, result on an optimistic assessment of the performance for most practical cases in which highly annotated strains from the same species as the test data or other closely related organisms are not available for training. In these figures we see drastically different results from those in Figure 12. In the sm-enzyme set, the gain from including the additional scores has disappeared and all combiners give similar performance, only slightly better than the best individual score. More notably, in the known-function set, most combiners are worse than the best individual score. Only the confidence-product combiner trained with the original plus the new scores does not show a degradation over the best individual scores. Furthermore, the decision tree combiner behaves worse than the confidence-product one. Due to its higher complexity, this combiner adapts more to the training data than the simpler one and, hence, when the test data does not follow the patterns observed in training, the performance is severely affected. Note that this behavior is not exclusive to decision tree combiners. Any combination strategy that involves a significant amount of parameters (like support vector machines with non-linear kernels) will be prone to overfitting of the training data. The confidence-product combiner, which has few parameters to learn, has a more stable performance, much more independent of the data used for training. On the other hand, as we have seen, also as a consequence of this lack of complexity this combiner is worse than the decision tree combiner when good training data is available.

The fact that the effect of the training data is much more marked when the known-function sets are used for training and testing instead of the sm-enzyme sets might be due to several factors. It is possible that the genome context scores on the known-function set of samples are less consistent across organisms than in the sm-enzyme set, making the learning of the combination function inherently harder. More likely, this behavior might be due to the fact that, as mentioned earlier, the gold standard is probably less reliable in the known-function set than in the sm-enzyme set. While ECK12 is a highly curated database, the rest of the organisms used in this paper have undergone much less curation, making the gold standard on these organisms lower quality since pathways or complexes that exist in the organisms might not appear in the database. For these organisms, it is reasonable to expect the quality of the labels to be better for genes that have been tagged as enzymes than for other genes which might not have been studied as much. Furthermore, it is possible that the kinds of functional relationships we are currently not considering in our gold standard (for example, interactions due to proteins transiently binding to each other or other kinds of functional relationships not captured by our labeling procedure, as explained in Section 4.1) correspond to a larger proportion of samples on the known-function set than in the sm-enzyme set. If, for either reason, the gold standard is indeed less accurate on the known-function set than in the sm-enzyme set, since the combiner is learning from these labels, we would expect the combiner to perform worse on the known-function set.

The implication of the results presented here is that combination results trained on a certain database might not generalize to organisms that are not well represented in this database. Furthermore, the benefits from using more complex combination procedures might be overestimated when training and testing on the same organism (or set of organisms). The only way to fairly assess whether a combination procedure will be able to generalize to unseen organisms that are not closely related to those available for training is to devise the training and testing databases in a way that is representative of the actual testing conditions. How distant to the test organisms the train organisms should be depends on how the combiner will be used. If the goal is to use the combiner on organisms for which no closely related organism is available for training, this same kind of criteria should be used to select the training data when trying to assess the performance of the combination procedure.

## 5 Conclusions

We present a systematic study of individual genome context methods and their combination, which we believe is needed to better understand how to optimize these techniques. The families of methods studied in this paper are the gene cluster, gene neighbor, gene fusion, and phylogenetic profile. These are the methods widely used in the literature and in publicly available databases [41]. We propose the use of normalization techniques for the genome context methods and show that it can produce large performance gains. We study the optimal parameters for each method and the effect that the reference list of genomes has on its performance. We also show the performance of the different methods for a set of bacterial organisms. Furthermore, we present a careful study of the effect that the training data has on a combination procedure used to merge all genome context scores into a single score.

In comparative experiments across different individual methods, we find that methods that compute summary measures of the distance between homologs of the genes in the target genome across a list of reference organisms, commonly called *gene neighbor methods*, lead, in most cases, to the best overall performance of all genome context methods. Although this result was observed earlier [4], we have demonstrated that it is true for any choice of reference organism list and for almost all organisms on which we tested. In absolute terms, performance of the genome context methods varies widely (up to a factor of two) across organisms, but generally their ranking does not, the gene neighbor method being invariably among the best. The gene cluster is, for some organisms, competitive with the gene neighbor method at low sensitivities. Phylogenetic profile methods are generally worse than gene neighbor methods, in many cases by a large margin. The gene fusion method is the worst across most organisms. Note that the gene fusion and gene cluster methods are qualitatively different from the gene neighbor and phylogenetic profile methods in that they can generate scores only for a small proportion of gene pairs. With this consideration, we believe that the gene neighbor method can be considered the overall best genome context method in the literature since it leads to the best performance on most organisms and is able to generate scores for all gene pairs in the target genomes.

Three of the four genome context method families rely on the extraction of homologs for the genes in the target genome. As commonly done in bioinformatics, sequence similarity is used as a way to detect homology. For this, a threshold on the BLAST E-value must be chosen. A thorough study of the performance of the different methods varying the value of this threshold indicates that the optimal value is in the vicinity of 10^-4^. While this same value was found to be optimal for a phylogenetic profile method in [17], our results indicate that this value is also approximately optimal for all other genome context methods.

We also show that the size and composition of the reference organism list has a significant influence on the performance of the genome context methods. Organism lists containing many organisms that are closely related to each other negatively affect the performance of some methods since they violate their independence assumption. Reference lists can be pruned to exclude highly related organisms using a clustering procedure resulting in relative gains on sensitivity of around 5%. Overall, the optimization of the E-value threshold and the list of reference organisms results in a gain of around 8% in the sensitivity of the gene neighbor method with respect to the system presented in [4], which uses an E-value threshold of 10^-10 ^and no pruning of the list of reference organisms.

Genome context scores suffer, as do many other scores from different statistical processing problems, from a bias effect: scores are affected not only by the characteristic we wish to detect (in our case, functional relationship), but by other characteristics that consistently affect the values of the scores for a certain group of samples. Score normalization methods aimed at compensating for this bias are in standard use in signal processing problems but have not yet, to our knowledge, been applied to genome context methods.

We implement two score normalization procedures borrowed from microarray analysis. We show that score normalization methods aimed at equalizing the distribution of scores across genes leads to large gains of as much as 40% on some genome context methods. A relative gain of around 25% is observed on the phylogenetic profile method that uses mutual information as the similarity metric between profiles. With this improvement, this normalized phylogenetic profile score is, we believe, the best performing in this family of methods since it outperforms a method introduced in [19], which has in turn been shown to outperform other, some very sophisticated and computationally complex, phylogenetic profile methods. Finally, results from combining the individual genome context scores testing on *E. coli K-12 *gene pairs are presented. Two combination approaches are compared: a simple approach that converts each individual set of scores into confidence measures and combines them with a simple nontrainable function, and a more complex approach that uses decision trees as combiners. We show that, when a cross-validation procedure is used for training, the decision tree combiner can greatly outperform the simpler combiner. In this case, large gains are obtained when three scores proposed in this paper, two of them normalized as explained above, are added for combination to the four original scores previously proposed in the literature. Specifically, when cross-validation on all known-function *E. coli K-12 *gene pairs is used for training and seven scores are used for combination, a gain in sensitivity of around 20% is obtained with respect to the best individual score given by a gene neighbor method. This gain can be compared to that obtained with the simpler combiner method when combining only the four original scores. This system is comparable to those used for the Prolinks paper [4] and in the STRING database [14,16,42]. For this system we find that the gains from combination on *E. coli K-12 *gene pairs with respect to the gene neighbor method are less than 4%. Hence, our system results in a gain with respect to the state of the art in genome context methods when well-matched data is used to train the combiner.

To our knowledge, combination performance has always been tested either by training the combiner parameters on the test samples (for example, [4,14]), or using a cross-validation procedure (for example, [7,11]). These procedures (particularly train-on-test) are expected to lead to an optimistic assessment of the gains that can be achieved from combination on organisms that are not well represented on the training data. In this paper, we explore the performance of the two combiners mentioned above when training data from organisms other than the target organism is used. We find that when organisms that are phylogenetically distant from the target organism are used to train the combiners both combination methods fail to give gains with respect to the single best individual score. Nevertheless, adding the normalized scores proposed in this paper seems to add some robustness to the procedure, allowing the simpler combiner to be at least as good as the individual best score.

Our conclusion is that, if genome context scores are to be used on organisms that are not well represented or phylogenetically similar to those available to generate a gold standard, and a single score needs to be generated for each gene pair, then either the single best score, the gene neighbor p-value, should be used by itself or a simple combiner (with few parameters) should be trained, preferably using the normalized scores proposed in this work. Using a complex combination procedure that leads to large gains on cross-validation experiments is likely to lead to suboptimal results on these unseen organisms.

## Authors' contributions

LF, JD and PDK decided research directions and discussed and analyzed results. LF carried out the experimental research and drafted the manuscript. JD and PDK revised the manuscript for technical content. All authors read and approved the final manuscript.

## Supplementary Material

Additional file 1 **Study of the effect of E and Q parameters**. In this file we show results on the effect of the parameters E (E-value threshold for inferring homology) on the gene neighbor, phylogenetic profile and gene fusion methods and Q (percent overlap for finding Rosetta Stones) on the gene fusion method. Click here for file
