# Supplementary material for: A systematic study of genome context methods: calibration, normalization and combination
Source: BMC Bioinformatics. 2010 Oct 1;11:493. doi: 10.1186/1471-2105-11-493 (PMC3247869; doi:10.1186/1471-2105-11-493)
Supplement: Additional file 1 — Study of the effect of E and Q parameters. In this file we show results on the effect of the parameters E (E-value threshold for inferring homology) on the gene neighbor, phylogenetic profile and gene fusion methods and Q (percent overlap for finding Rosetta Stones) on the gene fusion method. [file 1471-2105-11-493-S1.PDF]

Study of the effect of the E and Q parameters

Luciana Ferrer, Joseph M. Dale and Peter D. Karp*

Artificial Intelligence Center, SRI International, Menlo Park, CA

Email: Luciana Ferrer - lferrer@ai.sri.com; Joseph M. Dale - jmdale@gmail.com; Peter D. Karp*- pkarp@ai.sri.com;

*Corresponding author

Additional file for *A systematic study of genome context methods: calibration, normalization and combination* by L. Ferrer, J. Dale and P. D. Karp

In this document we show results on the effect of the parameters E (E-value threshold for inferring homology) on the gene neighbor, phylogenetic profile and gene fusion methods and Q (percent overlap for finding Rosetta Stones) on the gene fusion method.

1 Gene neighbor and phylogenetic profile methods

As explained in Section 2 of the main paper, the phylogenetic profile, gene neighbor, and gene fusion methods use homology information for their computation. In most implementations of genome context methods, two genes are assumed to be homologous if the sequence similarity E-value returned by BLAST is smaller than a certain threshold. Use of thresholds from 10^{-10} [1] to 10^{-4} [2,3] have been reported in the literature.

Figure 1 shows the sensitivity when choosing the top 1% and 5% samples as sorted by each system, for three of the phylogenetic profile methods and the three gene neighbor methods on the sm-enzyme gold standard set as a function of the E-value threshold. Results for the gene fusion method for varying values of E are shown in the next section in this document. The figure shows that larger values of E up to 10^{-4} give a consistent advantage over smaller values for all genome context methods shown. The largest value, 10^{-3}, is not consistently better than 10^{-4} for all scores. Hence, since 10^{-4} has been independently found in [3] to be approximately optimal for phylogenetic profile methods, we use this value for all experiments in the main paper.

2 Gene fusion method

The gene fusion method has two tunable parameters (see Section 2.4 of the main paper): the E-value threshold, E, and the minimum percent of overlap required between the query genes and the matching gene, Q. Figure 2 shows a comparison of ROC curves for different values of Q for this method when E is fixed to the value chosen above, $E = 10^{-4}$. In [1], a minimum overlap of 70% is enforced. In our results, we

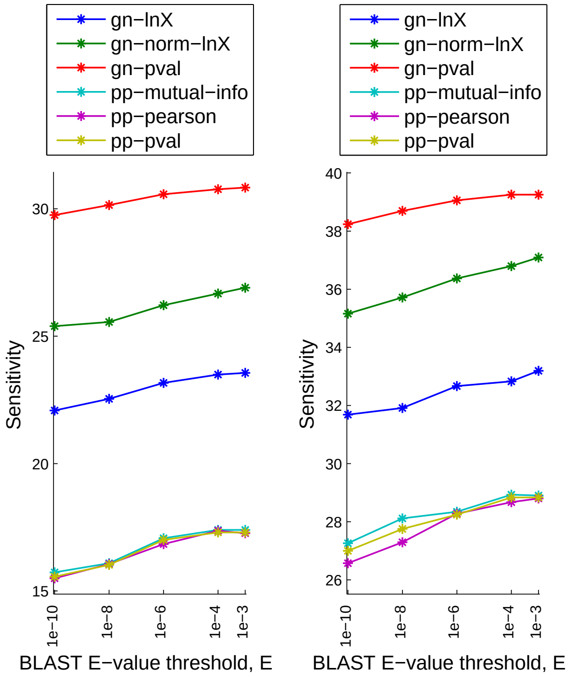

Figure 1: Sensitivity within the top 1% samples (left) and top 5% samples (right) for ECK12 on the sm-enzyme gold standard set as sorted by each genome context system, varying the BLAST E-value threshold, E, on a logarithmic scale. All three gene neighbor methods and three phylogenetic profile methods are shown. The full list of reference genomes is used to generate these scores.

see that smaller values of Q result in no degradation in performance while allowing for a larger coverage (indicated by the ROC curve reaching higher sensitivities). Specifically, the number of gene pairs that get a valid gf score for Q equal to 50 is 70% larger than when Q is equal to 70. For this reason, in the main paper we use $Q = 50$ for all experiments.

Figure 3 shows two ROC curves for the gene fusion method, both using the Q chosen from Figure 2 but with different values of E. Note that this figure, as well as Figure 2, show a much smaller corner of the ROC curve than the figures in Section 4.3.1 of the main paper. We see that a smaller E gives a slight advantage in the performance of the gf method at some operating points, but it also reduces the coverage. For this reason, and to be consistent with the value chosen for the full-coverage methods, we choose $E = 10^{-4}$ for any gene fusion experiment in the main paper.

References

1. Bowers P, Pellegrini M, Thompson M, Fierro J, Yeates T, Eisenberg D: **Prolinks: a database of protein functional linkages derived from coevolution**. *Genome Biology* 2004, **5(5):R35**.
2. Sun J, Xu J, Liu Z, Liu Q, Zhao A, Shi T, Li Y: **Refined phylogenetic profiles method for predicting protein-protein interactions**. *Bioinformatics* 2005, **21**(16):3409–15.
3. Sun J, Sun Y, Ding G, Liu Q, Wang C, He Y, Shi T, Li Y, Zhao Z: **InPrePPI: an integrated evaluation method based on genomic context for predicting protein-protein interactions in prokaryotic genomes**. *BMC Bioinformatics* 2007, **8**:414.

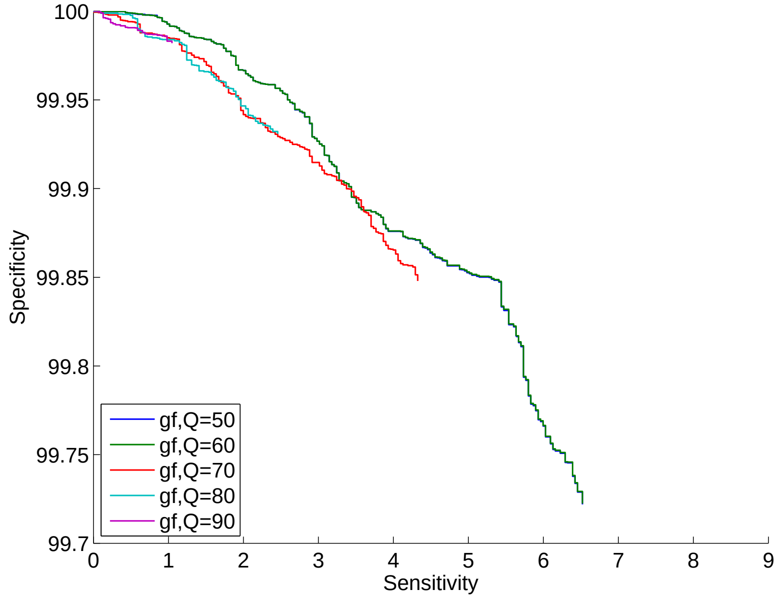

Figure 2: ROC curves on the sm-enzyme gold standard set for ECK12 for the gene fusion (gf) method using different values of Q, the minimum percent of overlap required between the query genes and the matching gene. The curve for $Q = 50$ is behind that for $Q = 60$. The value of E here is fixed at 10^{-4}. The full list of reference genomes is used to generate these scores.

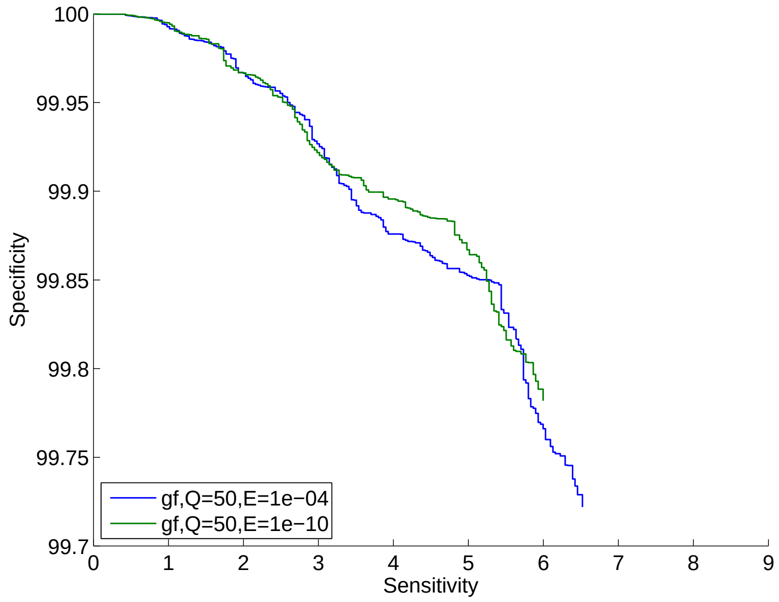

Figure 3: ROC curves on the sm-enzyme gold standard set for ECK12 for the gene fusion (gf) method using $Q = 50$ and two values of E, the maximum BLAST E-value. The full list of reference genomes is used to generate these scores.
